# Cross-Scale Molecular Analysis of Chemical Heterogeneity in Shale Rocks

**DOI:** 10.1038/s41598-018-20365-6

**Published:** 2018-02-07

**Authors:** Zhao Hao, Hans A. Bechtel, Timothy Kneafsey, Benjamin Gilbert, Peter S. Nico

**Affiliations:** 1 Earth and Environmental Sciences Area, Lawrence Berkeley National Laboratory, 1 Cyclotron Rd, Berkeley, California, 94720 USA; 2 Advanced Light Source, Lawrence Berkeley National Laboratory, 1 Cyclotron Rd, Berkeley, California, 94720 USA

## Abstract

The organic and mineralogical heterogeneity in shale at micrometer and nanometer spatial scales contributes to the quality of gas reserves, gas flow mechanisms and gas production. Here, we demonstrate two molecular imaging approaches based on infrared spectroscopy to obtain mineral and kerogen information at these mesoscale spatial resolutions in large-sized shale rock samples. The first method is a modified microscopic attenuated total reflectance measurement that utilizes a large germanium hemisphere combined with a focal plane array detector to rapidly capture chemical images of shale rock surfaces spanning hundreds of micrometers with micrometer spatial resolution. The second method, synchrotron infrared nano-spectroscopy, utilizes a metallic atomic force microscope tip to obtain chemical images of micrometer dimensions but with nanometer spatial resolution. This chemically “deconvoluted” imaging at the nano-pore scale is then used to build a machine learning model to generate a molecular distribution map across scales with a spatial span of 1000 times, which enables high-throughput geochemical characterization in greater details across the nano-pore and micro-grain scales and allows us to identify co-localization of mineral phases with chemically distinct organics and even with gas phase sorbents. This characterization is fundamental to understand mineral and organic compositions affecting the behavior of shales.

## Introduction

Shales are sedimentary rocks predominantly composed of fine grained mineral particles mixed with variable quantities of organic matter (OM), typically 5–20% w/w, including kerogen^1^. Shale rocks are effective traps and barriers for fluids, including methane and carbon dioxide (CO_2_), because the mineral and kerogen components form a low-permeability nanoporous network. Understanding the transport properties and reactivity of shale rock during oil and gas production, or during weathering reactions, requires knowledge of the nanoscale composition and mesoscale (nm–to–μm) heterogeneity. An adequate description of shale rock structure, mineralogy and chemistry, however, is currently beyond any single analytical or imaging approach^2^. Here we show that multiscale infrared spectroscopy and imaging, coupled to a neural network spectral classification algorithm, provides rich details of the chemical heterogeneity of shale.

Fourier transform infrared spectroscopy (FTIR) is a non-destructive method that can identify and quantify both mineral and organic substances. For example, FTIR analysis of bulk shale samples quantified the abundance of eight different minerals in shale samples in good agreement with X-ray diffraction analysis^3^. FTIR spectroscopy can also quantify measures of kerogen maturation. Specifically, the H/C ratio (A-factor) and O/C ratio (C-factor) derived from FTIR spectroscopy^4,5^ strongly correlate with elemental ratios obtained from the gaseous combustion method and the vitrinite reflectance value in a large number of shale samples^6^.

**Figure 1:**
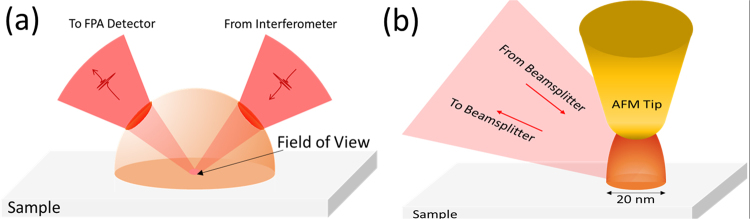
Diagrams of the setup of (**a**) the Germanium-hemisphere enhanced attenuated total reflection (Ge micro-ATR) and (**b**) the resonance enhanced SINS.

The long wavelengths of infrared light have traditionally limited the spatial resolution of the associated imaging method. Attenuated total reflection (ATR) FTIR microscopy can determine the mineral composition of a shale in two-dimensions but is limited to an approximately 100 μm spatial resolution^7^, and a recent study provides a considerably better characterization of the interconnected organic matter/mineral network, only limited to a 25 μm resolution^8^. An improvement of the ATR technique incorporates a large-radius germanium hemispherical crystal as the internal reflective element^9,10^ (Fig. 1a). The Ge hemisphere increases the numerical aperture of the micro-ATR technique and improves the spatial resolution by the index of refraction of the hemisphere, which is ~4 for Ge. We used the Ge micro-ATR technique coupled to a high-density focal plane array detector (FPA), which allows full spectral images with dimensions of 100 s µm and spatial resolution of detection approaching 1 µm without sample displacement or image distortion^11^.

An alternative and recently developed technique provides infrared imaging and spectroscopy with nanometer spatial resolution, nearly 1000 times smaller than the limit imposed by diffraction^12,13^. In this technique, a metallic atomic force microscope (AFM) tip enhances the intensity of infrared light at the near-field of the sample surface, and scattered light that has interacted with the sample is captured by an infrared detector (see Fig. 1b). After demodulating the detector signal at harmonics of the tip oscillation frequency, the near-field signal reveals information about the sample with tip-limited spatial resolution. When combined with a broadband synchrotron source in an asymmetric Michelson interferometer configuration, synchrotron infrared nano-spectroscopy (SINS) can span the mid- and far-IR^14^. Full spectral images are acquired by raster scanning over the sample surface which limits the throughput and applications, and as a result the 2D chemical image with nanometer resolution became available on a model sample just recently^15^. Although micro-ATR and SINS are both powerful approaches for microchemical analyses^11,16,17^, we show below that neither approach alone can provide mesoscale characterization of shale composition. Micron-scale spectroscopy does not permit unambiguous identification of nanoscale phases. Nanoscale spectroscopy cleanly resolves IR signatures of constituents but cannot generate chemical images at the necessary length scales. We show that a machine-learning method, in this case a single-layer neural network, can transfer spectroscopic information between techniques and hence across scales. We illustrate this approach with new insights into the distributions of organics, minerals and, surprisingly, gas-phase sorbents within a gas shale sample.

## Results and Discussion

### Infrared spectroscopy on bulk mineral standards and shale rock sample

**Figure 2:**
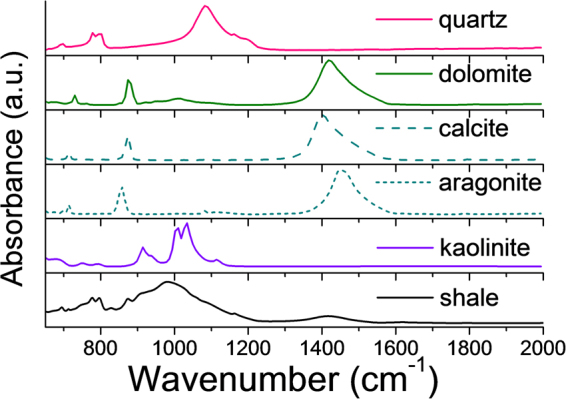
IR features of mineral standards and a shale bulk sample.

Figure 2 shows the infrared signatures from bulk standard minerals and the shale rock sample in this study. The infrared spectrum of the shale indicates it contains many reference minerals. For example, kaolinite features a strong Si-O stretching vibration at ~1010 cm^−1^ and a strong Si-O-Al stretch at 1110 cm^−1 ^^18^, while quartz features a single dominant peak at around 1090 cm^−1^ from Si-O asymmetric stretch^19^. Dolomite and related carbonates feature a strong peak at ~1450 cm^−1^ resulting from the asymmetric stretch of the CO_3_ anion^20^. Additional minor peaks at ~1720 cm^−1^, ~1650 cm^−1^, ~1400 cm^−1^ originated respectively from the carbonyl, aromatics and aliphatic groups from the kerogen^21^. However, the presence of multiple and overlapping spectral features in the bulk spectrum makes the confident identification of different minerals difficult.

### Ge micro-ATR FTIR of shale rock

**Figure 3:**
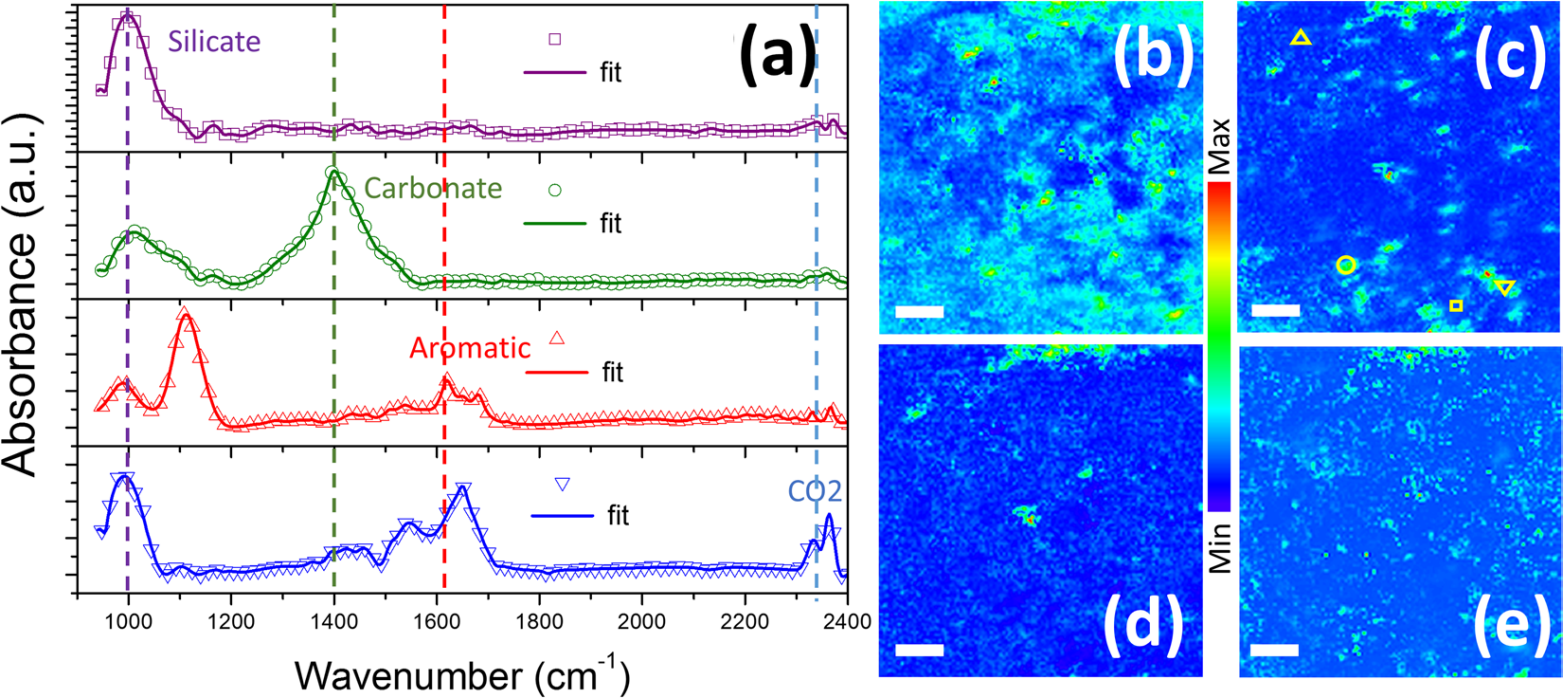
IR spectra and micrograph taken by the Ge micro-ATR method on the cross-sectioned shale rock sample. (**a**) 4 typical spectra showing features of silicates at ~1000 cm^−1^, carbonates at ~1400 cm^−1^, aromatics at ~1650 cm^−1^ and C-O stretches at ~2350 cm^−1^ from adsorbed CO_2_, indicated by the dash lines respectively. These spectra are selected from the locations marked with the corresponding symbols in (**c**). The solid curves are the best fit to the experimental data using KFit. **(b-d)** and (**e**) are reconstructed images from the peak areas at 1010 cm^−1^ (silicates), 1420 cm^−1^ (carbonates), 1300 cm^−1^ (aliphatics) and 1650 cm^−1^ (aromatics), respectively. The scale bars in (**b**–**e**) are 100 μm.

Clearer information on the content and distribution of organic and mineral materials is first obtained using the Ge micro-ATR method. Figure 3 shows 640 µm × 640 µm maps derived from FTIR spectra obtained from micron-scale areas in the cross-section of the shale rock and four spectra from selected locations. These maps (Fig. 3d,e) were reconstructed using the vibrational strengths for selected peak locations after a full-spectrum fitting with a multi-oscillator model. They highlight regions in which silicates, carbonates and organics contribute strong signals, as shown by the distinctive spectra (Fig. 3a) from selected locations within the mapped area. These micron-scale single-pixel data provide IR spectra in which individual contributions can be more clearly distinguished than in the bulk data. Notably, the broad silicate band at ~1000 cm^−1^ separates into distinct contributions from Si-O bonds in quartz and clays in these selected locations. In addition, different carbonates can be resolved through their contributions at 1390 cm^−1^ (calcite), 1410 cm^−1^ (dolomite) and ~1450 cm^−1^ (aragonite). Moreover, organic-rich area show contributions at ~1650 cm^−1^ (aromatic) and 1720 cm^−1^ (carbonyl) that were barely visible in the bulk data.

**Figure 4:**
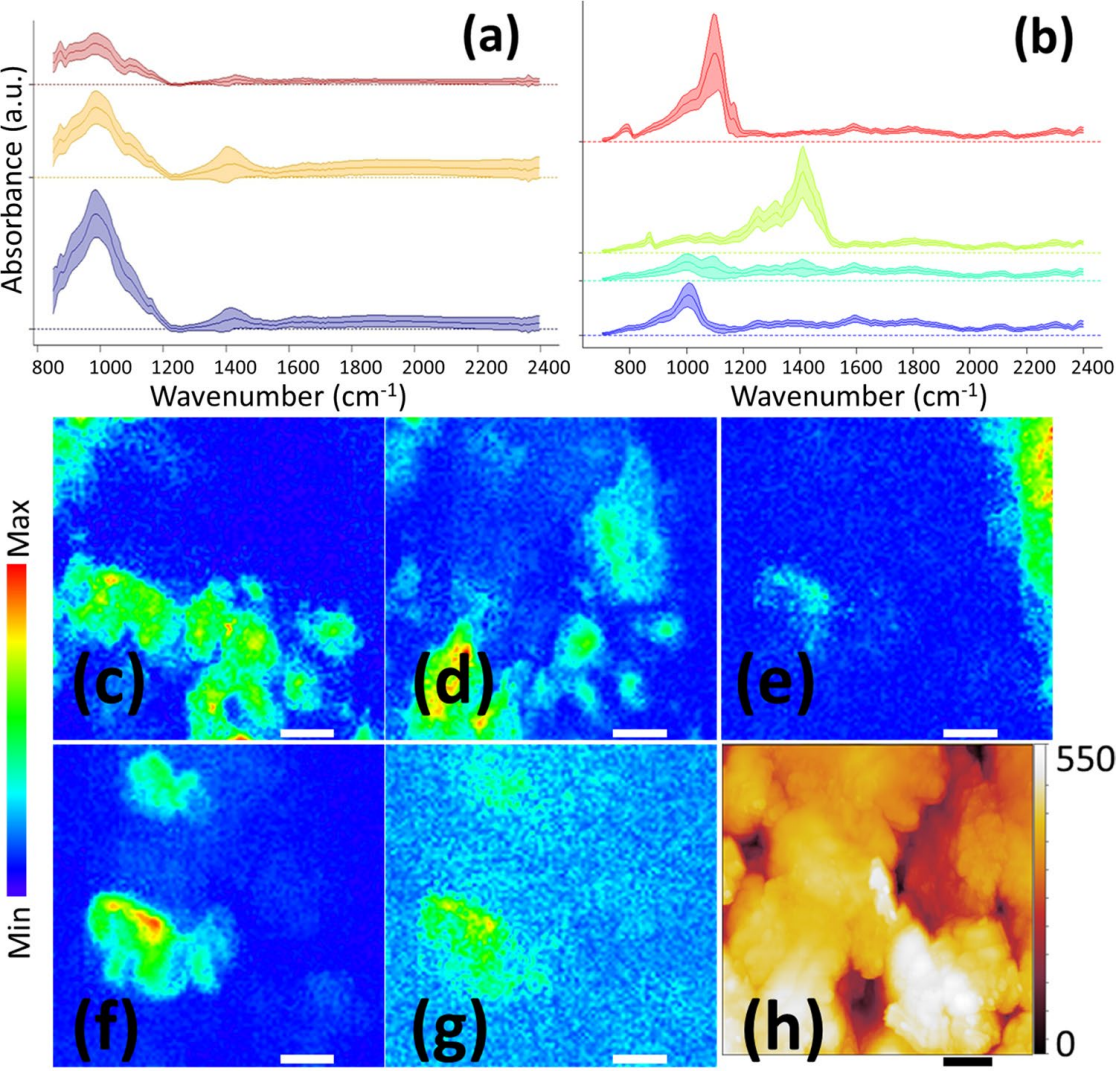
Top panel: (a) Grouping of IR data obtained from Ge micro-ATR method shows similar profiles because of the mixture of many features in the majority of the spectra, but they are grouped into distinctive groups with SINS spectra in (**b**). Bottom panel: Nanometer hyperspectral maps (**c**–**g**) with SINS. (**h**) Is the topography map of the same area as in the hyperspectral maps. The scale bars are 1 um. The maps are reconstructed from the absorbance peak areas at 1080 cm^−1^ for quartz (**c**), 1020 cm^−1^ for clay (**d**), 1450 cm^−1^ for carbonate (**e**), integrated strong scattering at around 1800 cm^−1^ for pyrite (**f**) and 1650 cm^−1^ for organics (**g**). There are visible “imprints” in (**g**) from the butterfly shapes in (**f**) as a result of the surface enhancement near the highly conductive pyrite surface.

It is difficult to evaluate the accuracy of the peak fitting method in many locations because the vibrational features are overlapped in most of the mapped area despite micron scale resolution. We sought to apply a standard spectral analysis method, principal component analysis followed by a k-means clustering to the full data set to identify the contributions from individual phases. However, this approach failed to extract distinct and meaningful components (Fig. 4a), because of the mixtures of phases overwhelmingly present within individual micrometer scale pixels.

### SINS imaging on shale rock

With the SINS method, much clearer mineralogical and chemical information is obtained. The SINS data (a total of 10,000 spectra) were successfully clustered and could be identified either as one of the mineral references (quartz, carbonate, clay), a strong-scattering class, or an organic-rich class. As illustrated in Fig. 4b, vibrational spectra obtained from single nanoscale pixels now show very close agreement with the data from standards, and the standard deviations of each group of the spectra are fairly narrow, much improved from the results of Fig. 4a. In particular, the SINS spectra allow weaker diagnostic vibrational modes to be detected, including the carbonate C-O out-of-plane bending (~880 cm^−1^, green curves) and the silicate symmetric Si-O stretch (~800 cm^−1^, red curves). We can now determine that the carbonate in this sample is mainly dolomite and that the silicate is quartz. Moreover, the precise location of the peak position at 1020 cm^−1^ (blue curves) indicates the likely existence of Mg-O-Si bonds. These conclusions could not be made from either the bulk or Ge micro-ATR data. There is also a group of spectra with signatures from organic chemicals, shown as cyan curves in Fig. 4b, which will be discussed in detail below.

The SINS data may show spectral evidence of spectral enhancement not observed in Ge-ATR data. First, SINS spectra from some areas contained no or few evident vibrational bands but were dominated by a very broad and strong scattering. This response is typically observed in SINS studies of conductive materials^22,23^ and hence we infer that these regions contain pyrite. Pyrite does not have any strong vibrational bands in the 800–3000 cm^−1^ range^24^ accessible by Ge or SINS and we did not include this mineral as a reference. Pyrite’s spectrum is therefore featureless using the Ge method, but the strong optical scattering signals in SINS were used for mapping this mineral. Note that since pyrite has distinct signatures at ~400 cm^−1^, a Si- or diamond-ATR method incorporating far-IR detection^25^ could be used to directly identify the pyrite content in future studies. The electric field enhancement at conductive pyrite surfaces may increase the vibrational signal from associated minerals and organics, and indeed reconstructed chemical maps show an apparent imprint of this enhanced signal, especially visible in the maps reconstructed from the generally weak organic peak (Fig. 4f). Modelling the full tip-sample interaction with recently developed models^26^ could be used to establish the full influence of this near-field enhancement effect.

In order to confirm our identification of all the minerals, we performed scanning electron microscopy (SEM) and energy-dispersive X-ray spectroscopy (EDX) on the same sample surface. The results are summarized in the Supplementary Figures 3 and 4. Although the EDX was not capable of detecting the molecular bonding information as we can with the infrared spectroscopy, the concentration of elements detectable in different locations confirmed the existence of pyrite, indicated by relatively enhanced emission signals of Fe and S co-localized at the same spots, carbonates (Ca and C), silicates (Si and O) and organics (C and O).

Second, energy transfer between closely–spaced vibrational bands can increase the signal from a weak resonance that lies on or near a dominant absorption^27^. Consistent with this mechanism, organic signatures in the 1200–1400 cm^−1^ region that lie close to the strong carbonate feature around 1450 cm^−1^ are overall stronger than for any other curve (Fig. 4b**)** while the organic signatures around 1650 cm^−1^ (used for mapping the organic distribution) are significantly weaker.

We constructed chemical maps using the vibrational strengths of selected peaks, and also mapped the high-scattering signal attributed to pyrite (Fig. 4c–f). The chemical maps show that even single morphological structures imaged by the SINS AFM tip (Fig. 4g) are of highly heterogeneous composition even within the mapped 1 μm area.

### Classification with neural network

**Figure 5:**
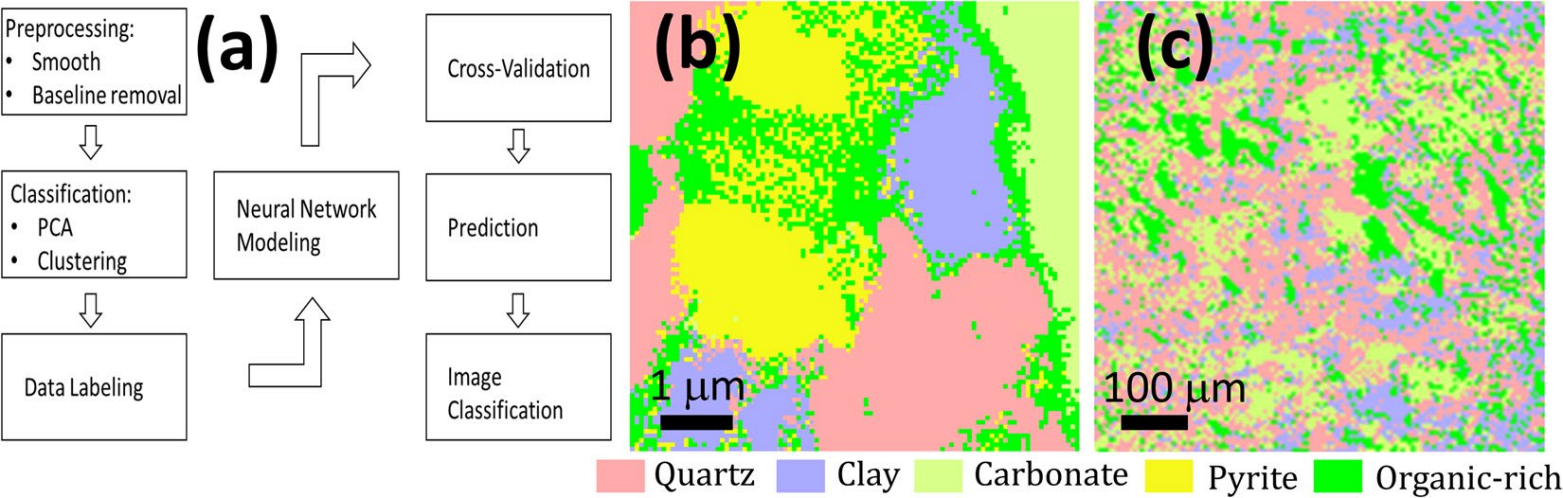
Diagram of the steps of performing neural network modeling and prediction (**a**) and predicted mineral classification map at nanometer scale (**b**) and micrometer scale (**c**). In the classification maps the pink corresponds to quartz, light blue clay, light green carbonate, yellow pyrite, and green organic-rich regions.

The chemical distribution maps of Figs 3 and 4 are not unbiased interpretations of the data because they are generated by intensities in manually selected peak regions that may contain contributions from different constituents. Moreover, the signal intensity varies significantly, likely due to surface topography, hiding chemical information from regions of low SINS scattering. For improved, unbiased data interpretation we developed a neural network (NNet) model. A large number of spectra in these blue areas included vibrational modes from organic molecules (*e.g*., 1300, 1350, and 1650 cm^−1^) as well as small peaks associated with silicate and carbonate (shown in the blue cluster in Figure S1); they are therefore classified as organic-rich. We used this data set, labeled as quartz, clay, carbonate and organic-rich, to train a 10-node NNet and then used the NNet to classify all the spectra in both the SINS for cross-validation and Ge data sets for prediction. We left out the pyrite class in the model because this phase is not detectable using the Ge micro-ATR method. The analysis flowchart is given in Fig. 5a and the predicted mineral distribution images for the nanoscale and microscale data are given in Fig. 5 b and c.

The map showing the classification of the SINS data provides a much more complete analysis of the shale distribution because is removes the intensity variations that are inevitable in the peak-fitting approach. Thus, the areas of low-intensity imaging (blue regions of Fig. 4c–f) are now fully interpreted. This approach also significantly improved the interpretation of the Ge dataset. Notably the chemical map of silicates (Fig. 3b) can be further classified into two maps of quartz and clay respectively in the classification map (Fig. 5c), and their matched locations further validate our model prediction in the microscale data. The classification procedure also helps to eliminate possibly misleading imprints from the near-field enhancement effect of conductive regions (Fig. 4c–f). Figure 5 shows that high-accuracy IR spectrum labeling and classification performed on a dataset with nanoscale spatial dimensions and resolution can be used to interpret a dataset with microscale resolution. This cross-scale molecular analysis method is not limited to IR spectroscopy of shale rock, but could be applied in the other many applications such as hyperspectral remote sensing or live cell imaging.

### Kerogen Distribution and Maturity

Two-dimension mineral distribution maps are commonly obtained for shale and other rocks using scanning electron microscopy (SEM) but this approach cannot obtain chemical information on the organic components^28,29^. Here we show that IR imaging combined with spectral analysis and classification can provide new insights into the relationships between mineral and organic components.

First, we observed evidence of the co-localization of organics with carbonate minerals. Although the Ge and SINS chemical imaging maps both display a highly heterogeneous distribution of organic functional groups, including OM not associated with any mineral phase, both approaches showed OM co-localized with carbonates more than any other mineral. This observation is in accord with the recent Raman/STXM study that found kerogen to associate with carbonates in a Mesozoic gas shale from northern Germany^30^.

**Figure 6:**
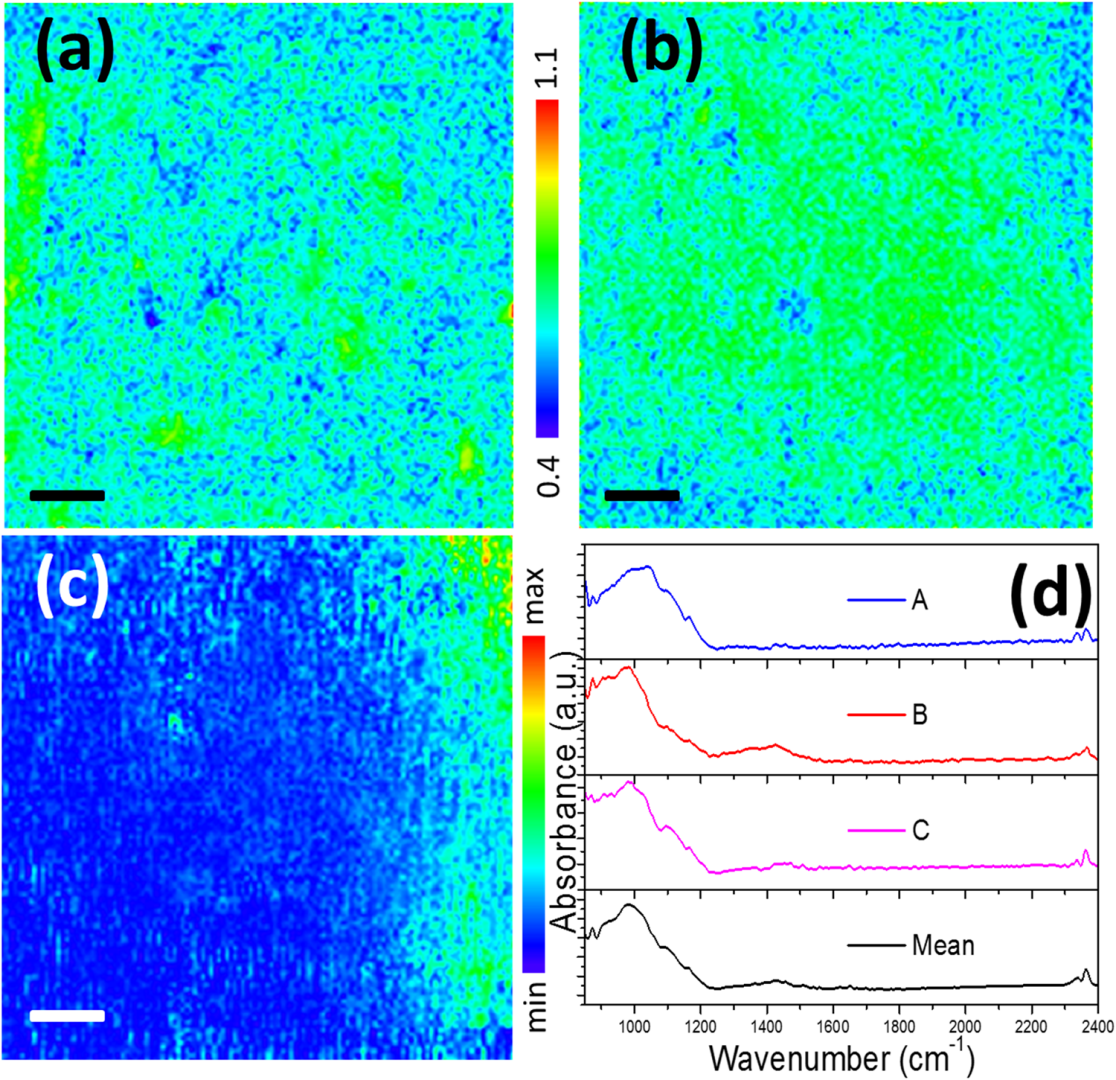
The C-factor (**a**) and A-factor (**b**) kerogen maturity maps derived from the Ge micro-ATR maps, with the same color scale from 0.4 to 1.0. (**c**) The distribution map of adsorbed CO_2_ derived from integrated peak area between 2300 cm^−1^ and 2400 cm^−1^. (**d**) Shows the randomly-chosen three spectra (A, B, C) and the mean spectrum of all the ~5400 spectra with “postive” CO_2_ content (black curve). The scale bars in the images are 100 μm.

Second, we observe substantial spatial heterogeneity in chemical signatures that have been used in bulk samples to determine kerogen maturity. The ratio of aromatic to aliphatic content (*i.e*., aromaticity) increases with maturity and with increasing carbon content in coals. Elemental ratios, such as the organic H/C ratio (called the A-factor) and the O/C ratio (C-factor) that quantify kerogen maturity can be measured directly through pyrolysis or indirectly through vitrinite reflectivity or FTIR analysis of bulk kerogen. In Fig. 6a and b we show possibly the first chemical distribution maps for IR-derived proxies for kerogen maturity at a micron scale. These maps show that bulk analyses provide averages over organic inclusions that can exhibit significantly different chemistry and apparent maturity. We find that kerogen with a high C-factor is selectively co-localized with carbonate minerals suggesting a link between organic matter oxidation, CO_2_ evolution and local precipitation of carbonate minerals. Further calculation using the averaged area values of corresponding peaks yields a C-factor of 0.26 and an A-factor of 0.70, which indicate the bulk material is a type II kerogen. This compares to the smaller values that we derived from the bulk measurement, 0.14 and 0.34 respectively, reflecting better resolved organic content because of the better resolved C-H and carbonyl bonds at high resolution (Figure S2).

### CO_2_ Distribution

Our micro-ATR analysis revealed a signal from molecular CO_2_ that was associated with the shale rock and not due to atmospheric interference. We generated a CO_2_ distribution map (Fig. 6c) using the absorbance strength from the antisymmetric CO_2_ stretching vibrations at 2350 cm^−1^ and 2360 cm^−1^. Over 5000 spectra in the map showed detectable CO_2_ absorption features in a somewhat preferred arrangement along the vertical direction of the image, with the mean of these and single-pixel examples given in Fig. 6d. This observation is in accord with recent studies^31–34^ showing that layered silicates in either controlled or natural settings, particularly in swelling clays, can adsorb or intercalate, and retain CO_2_. However, please note that this spatial correlation with CO_2_ is not at all unique, as we show in Fig. 3a, which suggests that the CO_2_ peaks correlates with the features of organics too. We did not observe any signal from methane in this gas shale, indicating CO_2_ to be higher affinity than CH_4_ to clay surfaces and interlayers. The infrared signatures of water which is important for shale heterogeneity are in general weak and narrow (e.g the O-H bending mode at ~1650 cm^−1^), or very broad (e.g. the O-H stretching mode at ~3300 cm^−1^) in this sample, as shown in Figure S5, so it’s difficult to retrieve its distribution map by fitting the peaks. However, by integrating the total absorbance in the frequency region related to the broadband O-H stretching mode, we were able to reconstruct a somewhat reasonable distribution map (right panel of Figure S5). In general the water concentration is fairly low in our sample, and concentrated in two confined regions. The potential contribution of the O-H bending mode to our observed infrared absorbance peak from organics (as we used in the SINS method) is therefore negligible as we show in the left panel of Figure S5.

## Conclusion

We used SINS to reveal the heterogeneous distribution of mineral and organic phases at nanoscale spatial resolution, and showed that a single-layer neural net that is trained using SINS spectroscopy can accurately classify spectra obtained from micron-scale analysis, thereby enabling representative rock areas to be characterized. This cross-scale molecular imaging methodology can be applied to shale rock and any other material with nanoscale organic and inorganic heterogeneity, and a multi-layer neural net, *e.g*. a convolutional neural network^35^, could be used to further quantify the pixel-level chemistry. The studies of a gas shale revealed kerogen maturity to be heterogeneous and show selective mineral associations, information on shale diagenesis difficult to obtain through any other non-destructive method. The Ge method will facilitate future studies of the influence of temperature and stress on the chemical and mineralogical structure of shale rock. Moreover, the ability to map the distribution of molecular CO_2_ adsorbed to clay minerals illustrates the potential for real-time studies of fluid interactions with nanoporous shale rock.

## Materials and Methods

### Samples

#### Shale rocks

We obtained Marcellus shale rock samples from a depth of 6306 feet in a gas-producing zone from a natural gas resource company. Using the infrared spectroscopic method, we determine that these shale samples are of the first type of kerogen with H/C ratio and C/O ratio around 1.2 and 1.1, respectively. The shale sample is rather soft in mechanical strength and we polished the surface across the cleaving lines, and the sample is then mounted on a metal plate by epoxy on the unpolished side.

#### Standard minerals and their reference spectra

Some of the mineral standards were obtained from Glenn Waychunas at Lawrence Berkeley National Laboratory. The others are a collection by BG at different field sites in North America. Some of them were powdered and those that were not were powdered by a tissuelyzer (Tissuelyzer II, QIAGEN) with a stainless steel ball at 1400 rpm for 10 minutes. The resulted fine powders of minerals were used as infrared standards for feature identification and extraction. The spectra of these mineral powders were obtained on a diamond ATR crystal in an infrared spectrometer (Nicolet IS50, Thermal Fisher Scientific Inc.) with 128 scans.

### Infrared Hyperspectral Image Data Acquisition

#### Ge-hemisphere enhanced imaging

A hemisphere of Germanium with diameter of one inch is used for near-field enhancement of infrared imaging. The infrared light from a globar infrared source is modulated by an interferometer, and then focused through a reflective 15× objective of a microscope (Cary 620 FTIR microscope and Cary 670 Spectrometer, Agilent Technologies) onto the top of the hemisphere. The 15× objective was used for imaging a large field of view, ~700 µm × 700 µm. The flat surface of the hemisphere is in contact with the solid shale sample, and the light from the microscope is further focused through the hemisphere to the flat interface. The reflected light from the solid sample is collected by the Ge hemisphere and then imaged back to a focal-plane array mercury cadmium telluride (MCT) detector (128 × 128 pixels) cooled with liquid nitrogen.

Because of the high refractive index of Ge, ~4, and enhanced collection of the evanescent waves at near-field, this setup allows us to image at very high resolution of around half of the wavelength of the light, ~1 to 4 micrometer in this study. By coupling to a FPA detector with 128 × 128 elements, we can increase the throughput by more than a thousand times than that with a conventional point detector. The further enhancement at near-field allows highly sensitive measurement on the heterogeneous surface on the shale rock sample, otherwise very difficult to collect due to the omnidirectional scattering light from grain boundaries with a far-field setup. During the measurement, the hemisphere’s flat surface is screwed tightly down to the polished surface of the shale sample. Since shale samples are soft in mechanical strength, we believe a very good contact had been maintained during the measurement, which can be proved by the overall satisfactory signal strength throughout the map.

A background image was first collected without sample contact, and then the infrared image of the sample surface was collected. Each image were averaged for 3 minutes, a truly high-throughput imaging method.

#### SINS

The SINS instrument at the Advanced Light Source (ALS) combines the brightness and spectral bandwidth of synchrotron infrared light with the sensitivity and spatial resolution of scattering type, s-SNOM to obtain broadband infrared spectra with AFM tip limited spatial resolution, which is typically less than 25 nm. The instrument is based on a customized atomic force microscope (AFM, Innova, Veeco Instrument Inc.) combined with a modified Fourier Transform infrared spectrometer (FTIR, Nicolet 6700, Thermo-Scientific) in an asymmetric Michelson interferometer configuration. Briefly, half the light from the synchrotron source is transmitted through a 50:50 KBr/Ge beamsplitter and focused on a conductive AFM tip with a parabolic mirror. The back-scattered light is collected by the same parabolic mirror and reflected toward an MCT detector by the beamsplitter. The other half of the incident light is directed toward the moving mirror in the FTIR bench and retro-reflected back to the beamsplitter and onto the detector. Movement of the reference mirror creates an interference signal on the detector and the corresponding interferogram is Fourier-transformed to extract the amplitude and phase spectra of the scattered signal. The AFM tip is operated in non-contact mode and the scattered signal is demodulated at twice the tapping frequency of the tip in order to extract the near-field signal from the far-field background. For the measurements presented here, an area was selected on the same sample that was scanned for the Ge-micro-ATR measurement. Hyperspectral maps were collected with 100 × 100 pixels over an 8 hour period with a step size of 64 nm to create an area map with total dimensions of 6.4 um × 6.4 um. Each pixel contains a broadband infrared spectrum from a ~20 nm spot, which is acquired at 16 cm^−1^ spectral resolution and averaged for 3 s. A template stripped gold surface was used to collect the background spectrum before mapping on the shale sample. The detailed data process afterward can be found in the reference^14^.

### Data Processing and Analysis

#### Spectral fitting

Quantification of spectral intensities in portions of the IR spectra was performed by fitting the full spectra with a series of Lorentzian peaks using the custom software, KFit Pro, written in C++ and compiled with Microsoft Visual Studio 2013. This multi-oscillator fit does not consider any reference spectra and allows images to be reconstructed based upon any fitted peak intensity.

#### Spectral classification

We used standard methods available in the R environment^36^ for classifying spectra obtained from the micro- and nano-IR. Following baseline correction and data smoothing^37^, we performed principal component analysis (PCA) and chose the top components that covered 98% or more over the data variance (typically 3 or 4). Subsequently, IR spectra from individual pixels were classified according to their similarity to the components by k-means clustering.

We use R environment for data analysis and machine learning. A number of R packages were used to perform baseline correction and data smoothing, principal component analysis, partial least squared regression (PLSR)^38^, and neural network (NNET)^39^.

#### Neural network model

The spectral classification of nano-IR data provided 4 chemically and mineralogically meaningful example spectra that clustered in distinct branches in PCA space (Figure S1). The data in the last group contained mostly organic and mixed mineral peaks, therefore they concentrated in the center of the PCA plots, and were not used for training to avoid overfitting but labeled as organic-rich in the reconstructed images. We used ~10,000 spectra that were labeled based on the reference data for unsupervised training of a neural network (NNet) model implemented in R. We composed a 10–node NNet with a single hidden-layer which allows us to run the model on a personal computer (16GB memory with one Intel i7-4790 CPU). The spectra were first reduced to contain only 10 uniformly spaced frequency bands by multi-point linear interpolation and then fed into the NNet model as inputs. The NNet quickly converged after ~40 iterations to a 100% accuracy rate of prediction achieved on the labeled data to within 10^−8^ relative tolerance.

The NNet outperformed alternative computational methods to classify spectra according to the principal components. For example, we evaluated the use of PLSR that identifies the dominant contribution of reference spectra to a new spectrum. The PLSR model built with 10 components only achieved 86% accuracy.

All the spectral data were then fed in the NNET model to be classified into different groups of minerals. We then render the classification data together at the corresponding peak positions into the reconstructed images, after the data were digitized into 32 bit and imported to imageJ software^40^.

#### Kerogen maturity analysis

We followed the approach of Ganz *et al*.^5^ who demonstrated that quantitative analysis of selected vibrational bands from FTIR data of bulk kerogen can provide a useful proxy for kerogen maturity, similar to a traditional van Krevelen diagram. Specifically, the A-factor was derived by finding the ratio of the peak area of C-H stretches (~2850, ~2920 cm^−1^) to the sum of this area and the peak area of the aromatic C=C bond (~1650 cm^−1^), and the C-factor was derived correspondingly by the ratio of the carbonyl (1710 cm^−1^) to the sum of the carbonyl and the aromatic C=C bond.

We calculated the Pearson’s correlation coefficients^41^ respectively between A- and C-factors, peak area of CO_2_ and the mineral classes identified by the NNet to find the significant co-locations described in the main text.

## Electronic supplementary material

Supplemental Information
