# Supplementary material for: Cross-Scale Molecular Analysis of Chemical Heterogeneity in Shale Rocks
Source: Sci Rep. 2018 Feb 7;8:2552. doi: 10.1038/s41598-018-20365-6 (PMC5803189; doi:10.1038/s41598-018-20365-6)
Supplement: Supplementary file 1 — Supplemental Information [file 41598_2018_20365_MOESM1_ESM.pdf]

Cross-Scale Molecular Analysis of Chemical Heterogeneity in Shale Rocks

Zhao Hao[1], Hans A. Bechtel[2], Timothy Kneafsey[1], Benjamin Gilbert[1], Peter S. Nico[1*]

[1] Earth and Environmental Sciences Area, Lawrence Berkeley National Laboratory, 1 Cyclotron Rd, Berkeley, California 94720

[2] Advanced Light Source, Lawrence Berkeley National Laboratory, 1 Cyclotron Rd, Berkeley, California 94720

* Corresponding Author; psnico@lbl.gov

SUPPLEMENTARY FIGURES

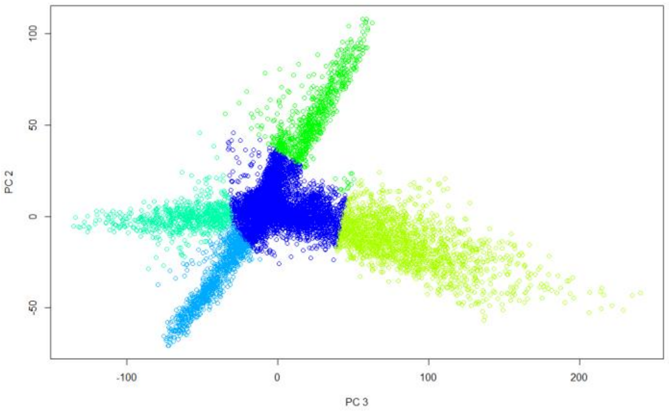

Supp. Fig 1: K-means clustering of the second and the third principle components from the PCA of the IR spectra obtained with SINS, showing distinctive differences between the infrared features.

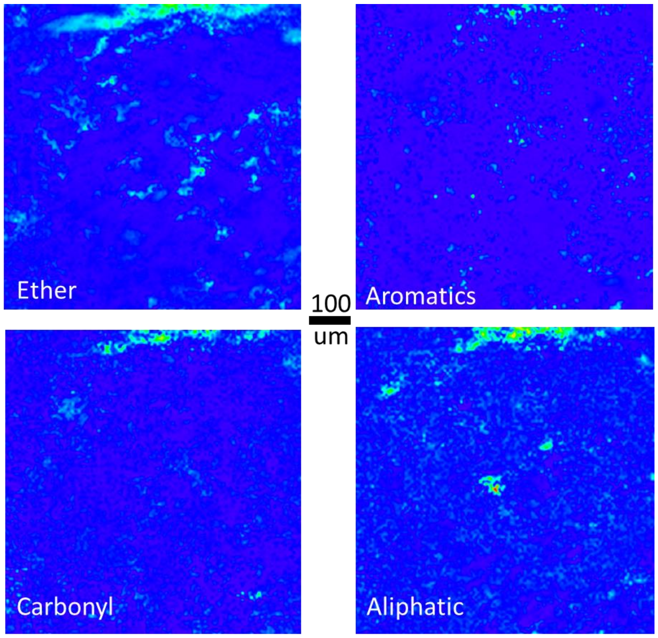

Supp. Fig 2: Reconstructed microscopic images from all the OM related peaks.

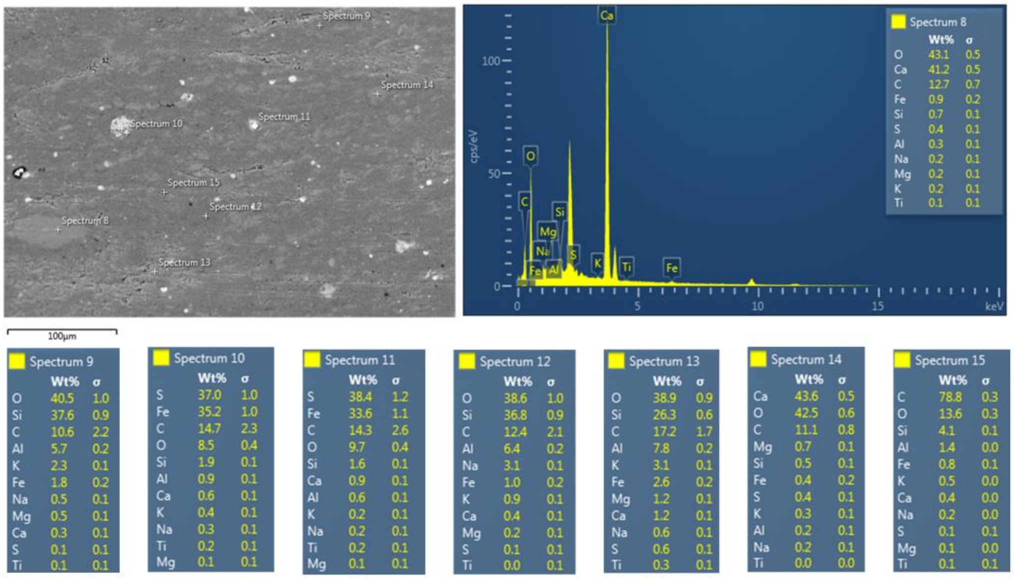

Supp. Fig 3: The upper-left image is an SEM micrograph of the surface from the same sample, but possibly not in the same region as in the infrared measurements. An EDX spectrum is shown in the

upper-right image, and the derived concentrations of detectable elements are shown in the following panels, indicating from left to right existence of silicates, sulfites, sulfite, silicate, silicate, carbonates, and organics at corresponding locations marked in the SEM micrograph. The scale bar under the SEM image is 100 μm.

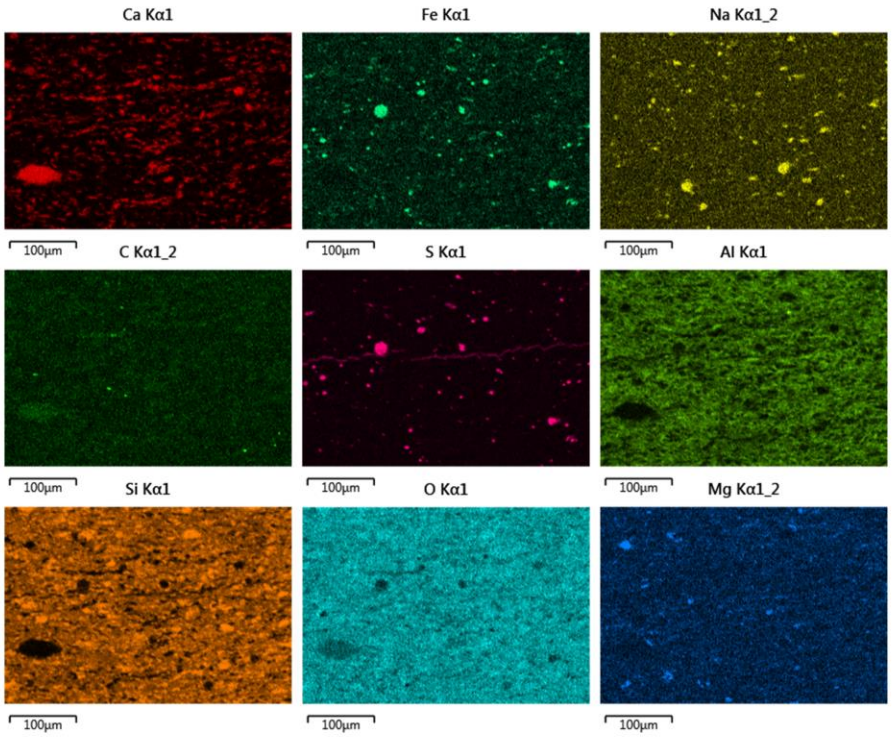

Supp. Fig 4: the chemical maps reconstructed from EDX spectra to confirm our mineral identification and prediction obtained from infrared imaging tools.

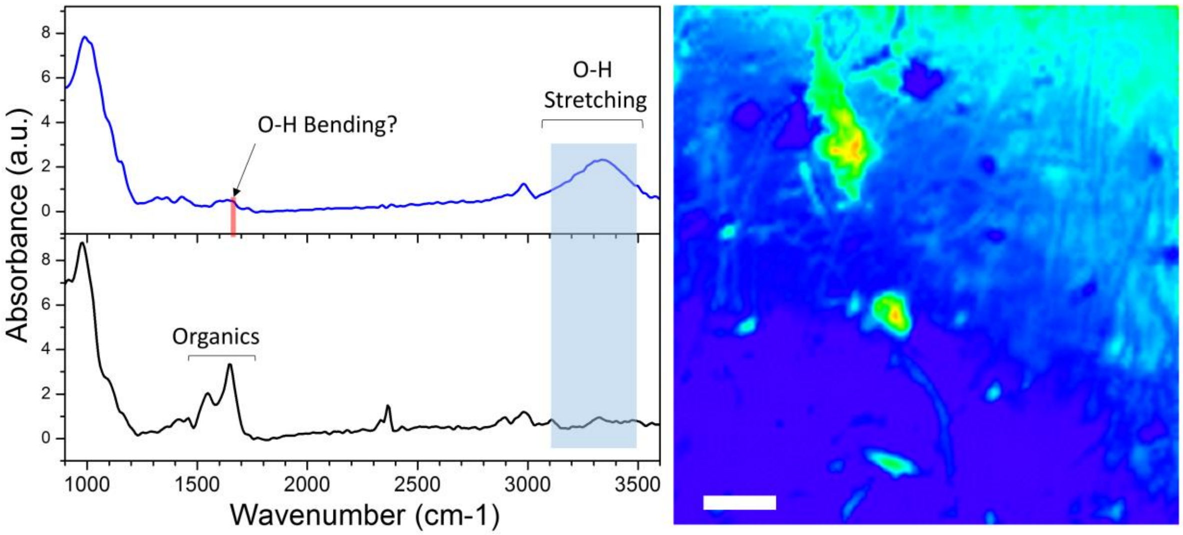

Supp. Fig 5: (left) the spectra with O-H signatures and organic signatures. The blue curve in the upper panel indicates the possible but very small contribution of O-H bending mode to the organic peak at 1650 cm^{-1} (red shaded region), as we used in reconstructed SINS image, Fig. 4g. (right) a reconstructed H2O distribution in our shale sample, obtained by integrating the O-H stretching mode from 3100 cm^{-1} to 3500 cm^{-1} (blue shaded region) from the Ge-Hemisphere imaging. Note that the integrated O-H stretching peak could include the contribution from organics, and sometimes baseline distortions because of the broad nature of this peak. The scale bar is 100 μm.
